# Brachytherapy reirradiation for local recurrences of gynaecological cancers: two clinical cases, literature review and OAR constraints

**DOI:** 10.3332/ecancer.2026.2091

**Published:** 2026-03-12

**Authors:** Javier Faundez, Carlos Schananier, Cynthia Villegas, Javier Retamales

**Affiliations:** 1 Instituto Nacional del Cancer, Santiago 8380455, Chile; 2 Facultad de Medicina, Universidad de Chile, Santiago 8380453, Chile; 3 Hospital Sotero del Rio, Santiago 8207257, Chile

**Keywords:** brachytherapy, salvage treatment, gynaecological cancer, narrative review

## Abstract

**Background:**

Local recurrence of gynaecological malignancies after prior pelvic radiotherapy presents a complex therapeutic dilemma. Pelvic exenteration is curative in select cases but carries high morbidity and profound quality-of-life impact. Salvage reirradiation with image-guided brachytherapy (BT) offers a highly conformal alternative; however, data remain fragmented and practical organ-at-risk (OAR) constraints are not standardised.

**Objectives:**

1) To describe in detail two contemporary cases of high-dose-rate (HDR) BT reirradiation – one vulvar and one vaginal vault recurrence – highlighting planning strategy, catheter implantation, dosimetry and clinical outcome; 2) to synthesise current evidence on efficacy, toxicity, dose–fractionation and technique of BT reirradiation in cervical, endometrial and vulvar cancers; and 3) to propose pragmatic cumulative EQD2 OAR limits for rectum, bladder, small bowel and urethra based on published series.

**Methods:**

Both patients underwent computed tomography/magnetic resonance imaging-based contouring with hybrid interstitial or surface mould applicators. Total doses were 45.6 Gy in 8 fractions (EQD210 ≈ 60 Gy) for the vulvar recurrence and 24.6 Gy in 3 fractions (EQD24.5 ≈ 48 Gy) for the vaginal vault recurrence, with cumulative OAR doses referenced against literature-derived thresholds. A narrative review (1993–2024) identified 35 key studies involving >600 reirradiated patients.

**Results:**

The vulvar case achieved complete response at 6 months without Grade ≥3 toxicity; the vaginal vault case showed initial partial response, remained ECOG 0 and required systemic therapy for later progression. Across the literature, 1- to 3-year local control ranged 40%–80% and overall survival 40%–60%, with severe late toxicity generally ≤30% when cumulative doses respected D2cc ≈ 70–100 Gy3 (rectum), 90–120 Gy3 (bladder) and D0.5cc ≤ 110 Gy3(urethra). Fractionation of 4–6 Gy BID for 5–10 fractions (EQD210 ≈ 40–50 Gy) was most common.

**Conclusion:**

HDR BT reirradiation can achieve meaningful, sometimes durable, local control in carefully selected gynaecologic-cancer recurrences while preserving pelvic organs. Adhering to proposed cumulative EQD2 OAR constraints and employing image-guided interstitial techniques are paramount to curtail severe complications. Multidisciplinary assessment and treatment at experienced centres are strongly recommended to optimise outcomes.

## Introduction

Local recurrences of gynaecological malignancies – particularly cervical, endometrial and vulvar cancers – pose significant therapeutic challenges. Patients frequently have undergone extensive treatment, including radical surgery, external beam radiotherapy (EBRT) and sometimes chemotherapy. For many, the recurrent disease is confined to the central pelvis or vaginal vault. The traditional salvage procedure for central pelvic recurrence has often been pelvic exenteration. While this surgery may achieve local control, it is associated with high rates of morbidity and a profound impact on quality of life [1–3].

An alternative salvage approach is reirradiation, potentially using brachytherapy (BT). BT can deliver high, localised doses to the recurrent lesion while limiting radiation exposure to surrounding healthy structures. This targeted dose distribution is especially valuable in previously irradiated patients, for whom tolerance of normal tissues is often the limiting factor.

Although reirradiation with BT is not novel – historical case series date back decades [4] – its routine incorporation into practice has been slower, in part due to limited awareness, lack of training in advanced implant techniques and concerns about toxicity. Nonetheless, increasing availability of modern imaging computed tomography (CT) and magnetic resonance imaging (MRI) for treatment planning and advanced applicator systems has refined BT’s precision, making salvage reirradiation a viable option for many patients who may not tolerate or be candidates for surgery.

This article reports two clinical cases of salvage reirradiation in recurrent gynaecological cancers. It also summarises relevant literature that examines outcomes, toxicity*, dose considerations and technical aspects of image-guided brachytherapy for reirradiation. While the data are largely drawn from retrospective series, results suggest that reirradiation with BT can lead to substantial rates of local control – sometimes exceeding 50% – with acceptable late toxicity (mostly ≤ Grade 2), especially when delivered at experienced centers.

*All toxicities reported in this article are graded according to CTCAE v6.0

## Case 1

### History and presentation

An 86-year-old woman with a history of atrial fibrillation on direct oral anticoagulation presented initially with a vulvar tumour that extended across the left labium majus, the upper third of the right labium majus and the clitoris. Physical exam revealed a 3 cm fixed left inguinal node. A staging CT scan demonstrated diffuse thickening of both labia majora and a 3.4 cm left inguinal lymph node. Biopsy confirmed a moderately differentiated infiltrating squamous cell carcinoma, leading to a diagnosis of Stage IIIB vulvar cancer.

She received neoadjuvant EBRT (total of 47 Gy to the vulva and regional nodes and 58.8 Gy as a simultaneous integrated boost to the inguinal node) plus weekly cisplatin. Three months after completion, she underwent a radical vulvectomy and bilateral inguinal lymphadenectomy, with pathology showing no residual disease (pathologic complete response).

After more than 2 years of follow-up, a recurrent 4 × 2 cm vulvar tumour appeared at the labia majora and clitoris, sparing the urethra, anus and perineum. MRI suggested tumour abutting the distal urethra. A restaging CT showed no metastatic disease. As salvage surgery was deemed infeasible by the gynaecologic oncologist, the patient was referred for reirradiation with interstitial BT.

### Treatment planning and procedure

**Figure 1.**
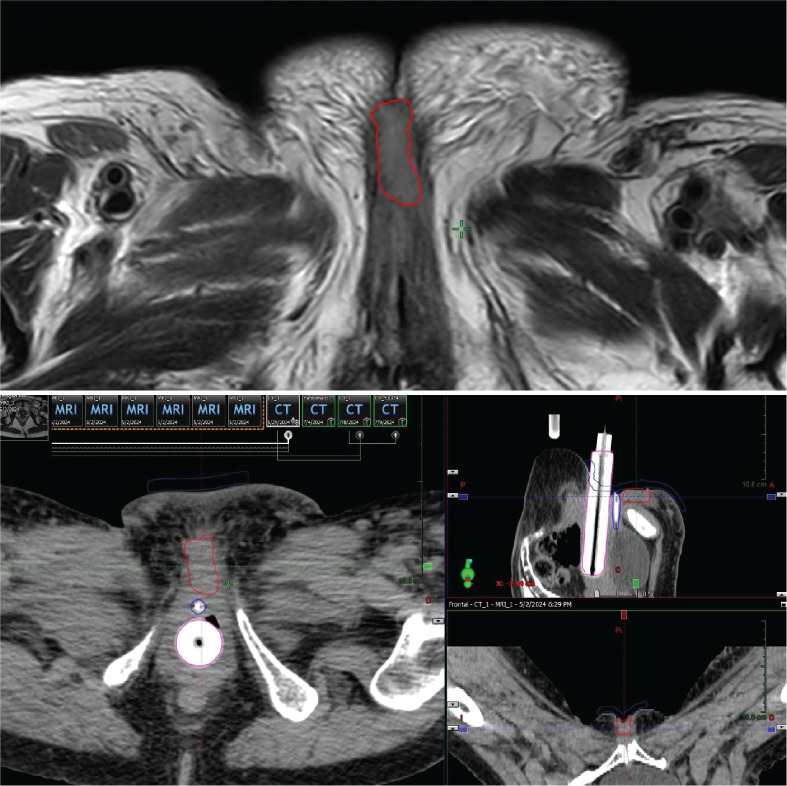
Case 1. Above, MRI of the pelvis, T2 sequence. The red line shows the GTV. Below, CT simulation for pre-plan design, on the axial, coronal and sagittal axis. The GTV is shown in red, the urethra in blue and the vaginal cylinder in pink.

A key step was a detailed clinical exam to determine the extent of disease and the feasibility of placing interstitial catheters at a safe distance from the urethra. A planning CT (2.5 mm slices) was fused with a diagnostic MRI to precisely delineate the gross tumour volume (GTV) (Figure 1).

Because the patient had previously received 47 Gy to the vulva (plus a higher boost to the lymph node area, which was anatomically separate), the oncologist and dosimetrist considered the dose already delivered to local organs at risk (OAR). The urethra, in particular, required careful attention. A common threshold is to maintain a cumulative dose of roughly 70–120 Gy EQD2_3 for the urethra, depending on the guidelines used [5].

In the pre-planning phase, an 8-fraction schedule (5.7 Gy/fraction) was proposed, for a total biologically effective dose of about 59.7 Gy EQD2_10. A cylinder placed in the vaginal canal was initially considered, but imaging showed that the recurrent vulvar lesion lay at a distance such that the cylinder would not effectively contribute dose. Instead, the plan required six to seven interstitial catheters.

**Figure 2.**
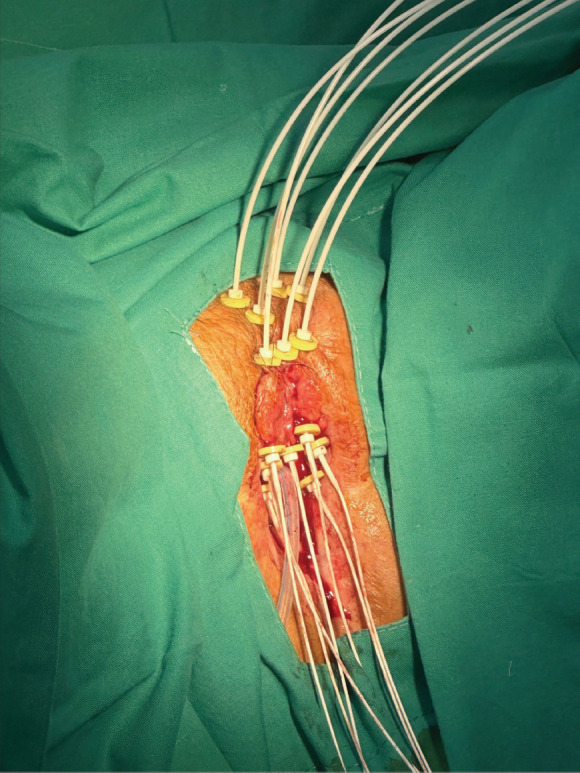
Case 1. The vulvar tumour and the implantation of seven interstitial catheters are observed.

Under spinal anesthesia, the implant procedure involved placing three flexible catheters on each side of the urethra at different depths (roughly 1 cm between each), plus an additional superficial catheter in the central region of the tumour. The catheters were secured with small radiopaque fixation buttons (Figure 2). A post-implant CT scan was performed to confirm the positioning of the catheters relative to the GTV and OAR.

**Figure 3.**
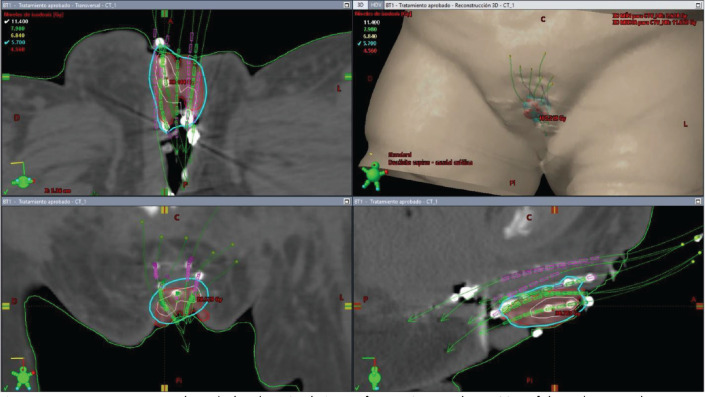
Case 1. 3D treatment plan calculated on simulation CT for fraction #1. The position of the catheters and the dosimetric distribution in the axial, coronal and sagittal planes are observed, in addition to a 3D reconstruction. In light blue, the isodose of 5.7 Gy; in white, the isodose of 11.4 Gy.

**Figure 4.**
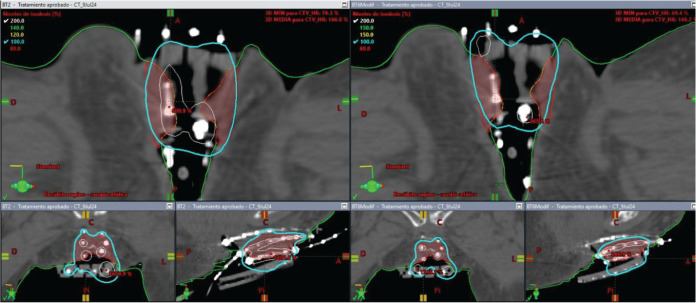
Case 1. 3D treatment plan with bolus. The position of the catheters and the dosimetric distribution in the axial, coronal and sagittal planes are observed. On the left, fraction #2 to #5. On the right, fraction #6 to #8. In light blue, the isodose of 5.7 Gy; in white, the isodose of 11.4 Gy.

The initial plan suggested possible undercoverage in superficial regions of the tumour (Figure 3). Therefore, a custom silicone bolus containing four additional catheters was placed on the vulvar surface to enhance coverage of peripheral edges. A repeat CT confirmed improved D100 and D90 of the GTV while maintaining urethral dose constraints (Table 1, Figure 4).

### Outcomes and follow-up

Treatment was delivered over 1 week, with the patient hospitalised. She received eight fractions of high-dose-rate (HDR) BT (using an Ir-192 source). The urethra’s partial volumes stayed well below the established dose limit. Mild vulvar pain (grade 1) and grade 1 radiodermatitis were the main acute toxicities; both resolved within weeks. No acute or late toxicity was observed involving the rectum, bladder, small bowel or urethra. Toxicities were graded according to CTCAE v5.0 [30].

**Figure 5.**
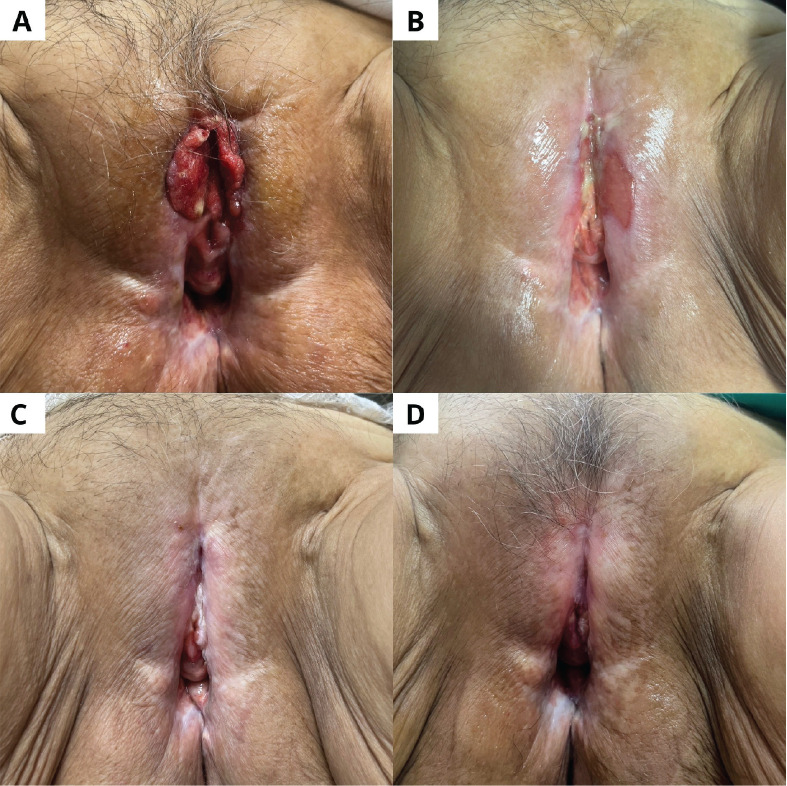
Case 1. (a): Vulvar tumour prior to treatment. (b): 6 weeks later. (c): 3 months later. (d): 6 months later.

At 3 months, MRI and physical exam showed no evidence of residual disease. Six months post-treatment, she remained asymptomatic (ECOG 0), with no sign of local recurrence (Figure 5).

## Case 2

### History and presentation

**Figure 6.**
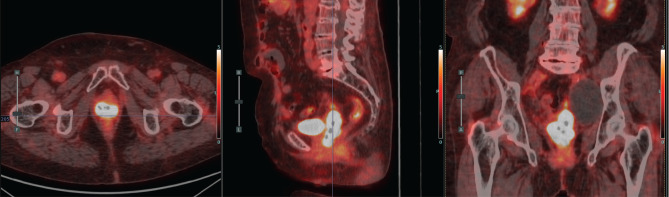
Case 2. PET-CT scan prior to BT. Axial, sagittal and coronal section. A hypermetabolic tumour mass in the vaginal vault and a left pelvic lymphocele were observed.

A 75-year-old with a history of hypertension and dilated cardiomyopathy was diagnosed with Stage IIIC2 endometrial adenocarcinoma after hysterectomy, bilateral salpingo-oophorectomy and lymph node dissection revealed one positive para-aortic node. She declined or missed timely adjuvant radiotherapy. Seven months after surgery, the patient presented with a symptomatic recurrence in the vaginal vault and a positron emission tomography - computed tomography (PET-CT) also revealed latero-aortic hypermetabolic adenopathy. She received letrozole for hormone receptor–positive disease and six cycles of palliative chemotherapy with Carboplatin – Paclitaxel, after which she had complete regression of the adenopathy and stability of the disease in the vaginal vault. Then she was treated with palliative EBRT (30 Gy in 10 fractions) to the pelvis and vaginal vault. One year after this treatment, she developed progression of local disease involving the distal vagina and possibly the urethra (Figure 6). The case was discussed in tumour board, and pelvic exenteration was considered but deemed too morbid. She was referred for HDR interstitial BT reirradiation.

### Planning and implant

Because her tumour extended within the entire vaginal canal, with possible urethral involvement, a hybrid endocavitary–interstitial approach was chosen. A custom 3D-printed template was designed with multiple channels to guide needle insertion transperineally, along with a central opening for a 2 cm cervical applicator.

**Figure 7.**
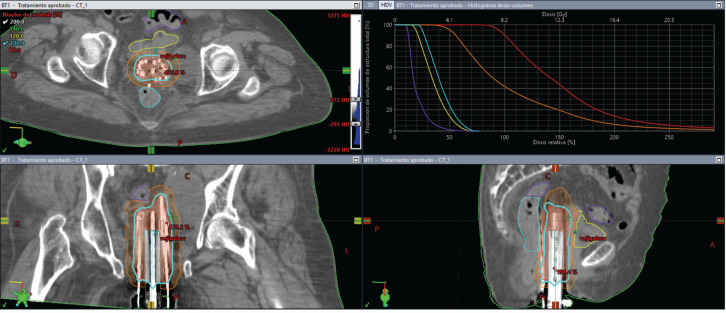
Case 2. Dose-volume histogram and dosimetric distribution of fraction #1 in axial, coronal and sagittal planes. It is shown in red CTV-HR, in orange CTV-IR, in light blue the rectum, in yellow the bladder and in purple the sigmoid colon.

On the day of implant, the patient underwent spinal anesthesia, and a Foley catheter was placed. The custom template was positioned against the perineum, and 18 interstitial needles were inserted according to the pre-plan. An initial fraction was delivered at 8.2 Gy to the 90% isodose of the GTV (Figure 7). For subsequent fractions, the central channel was exchanged for a deeper needle to cover more cranial disease.

Total dose was 8.2 Gy × 3 fractions, equating to roughly 48.1 Gy EQD24.5. The plan accounted for prior EBRT (30 Gy in 10 fractions). Although the bladder, rectum and sigmoid colon had some overlap with the new target volume, no dose-limiting constraints were exceeded (Table 1).

### Outcomes and follow-up

The patient developed transient Grade 2 urinary irritative symptoms (frequency and dysuria) that resolved within 4 weeks post-treatment. No rectal bleeding, bowel changes or urethral discomfort were reported. No late Grade ≥2 toxicities involving the rectum, bladder, small bowel or urethra were documented. All toxicities were assessed and graded per CTCAE v5.0. Four months later, a PET-CT showed a slight decrease in tumour size and metabolism in the vault and proximal vaginal canal, measuring 40 mm (versus 48 mm on the pre-treatment PET-CT) and an SUVmax of 11 (versus 18). Because of this residual tumour activity, the patient received a second course of palliative chemotherapy with Carboplatin – Paclitaxel for six cycles. New images showed local and systemic progression. Doxorubicin was started then. Nonetheless, from a performance standpoint, she remained ECOG 0 after BT, capable of normal daily activities. Twenty-one months post-BT, she continued palliative treatment with no major local complications attributed to reirradiation.

### Literature review

BT allows very focal dose escalation in sites where EBRT alone might expose the bowel, bladder or other pelvic structures to unsafe levels [4, 6]. A recurrent lesion often lies in the central pelvis or vaginal vault. If surgery is not feasible, reirradiation is one of the few remaining curative-intent options.

While randomised trials on reirradiation in gynaecological cancer are extremely scarce, numerous retrospective series underscore the feasibility and effectiveness of this approach in carefully selected patients. Overall local control rates vary between 40% and 80% depending on tumour size, prior dose, site of recurrence and technique [7–9].

In a prospective phase II trial, Martínez-Monge *et al* [7] analysed 50 patients with locally advanced and recurrent gynaecological cancer – 25 of whom had received prior irradiation. In patients without previous radiation, the treatment approach consisted of neoadjuvant chemoradiotherapy followed by surgery and HDR BT, whereas in those who had already been irradiated, surgery was combined with HDR BT. Long-term follow-up (median 11.5 years) demonstrated a local control rate of 59.6% at 14 years among the reirradiated cohort, although dose reductions became necessary to reduce Grade ≥3 toxicities. Another study by Amsbaugh *et al* [8], retrospectively evaluating 21 patients with recurrent gynaecologic cancers who underwent interstitial BT (median total dose of 22.5 Gy in HDR or approximately 41.5 Gy in low-dose-rate (LDR)), noted a 1-year locoregional failure-free rate of roughly 71.5% and an 82.2% 1-year overall survival (OS), with Grade 3 or higher vaginal, urinary or rectal toxicities staying below 30%. Similarly, Kellas-Ślęczka *et al* [9] assessed interstitial HDR BT for 14 patients with locally advanced or recurrent vulvar cancer and observed favourable local control rates alongside minimal Grade 3–4 toxicities. A broad review by Sturdza *et al* [10] (published under the auspices of the American BT Society working group), examining reirradiation for cervical and endometrial cancers, underscored that BT alone or in conjunction with a more limited EBRT field can offer curative reirradiation for small recurrences, provided normal tissue constraints are respected.

Focusing on recurrent endometrial cancer, Ling *et al* [11] documented a 66% 3-year local control rate in 22 patients reirradiated with three-dimensional BT, with or without EBRT; many of these individuals had previously received either EBRT or vaginal BT. Huang *et al* [12], investigating a cohort of 16 patients with recurrent endometrial cancer, reported that individualised reirradiation doses based on risk categories yielded a 53% local control rate at 2 years, although four patients experienced some degree of toxicity. In the context of recurrent cervical cancer, Mahantshetty *et al* [13] evaluated 30 patients who were reirradiated using interstitial or intracavitary HDR BT, finding a 2-year local control rate of 44% overall but 52% in those receiving higher doses (exceeding 40 Gy EQD2). Likewise, Mabuchi *et al* [15] examined a group of 52 patients with central recurrences who were administered 6 Gy × 7 fractions, noting a 77% clinical response at 2 months, though around 25% of patients experienced severe toxicity. LDR or permanent implants, such as 198Au or 131Cs, have also shown promise, with local control rates of about 70%–85% for small or superficially located recurrences [16–19].

Regarding dose and fractionation regimens, HDR BT frequently employs 4–6 Gy per fraction administered twice daily, targeting an overall EQD2_10 of 40–50 Gy or more, though once-weekly high-dose treatments have also been described. The decision on fractionation schedule typically hinges on tumour size, OAR proximity, previous radiation dose and the time elapsed since earlier radiotherapy. Some centers opt for more conservative total doses – around 24–32 Gy – to curtail severe toxicity, but there is a potential reduction in local control if the total dose is insufficient [7, 10]. In certain cases, stereotactic body radiotherapy (SBRT) is used for pelvic reirradiation, but substantial toxicity, such as bowel injury or fistula formation, remains a concern in extensively pretreated fields [20, 21]. If prior irradiation was restricted to the vaginal vault, external beam reirradiation can sometimes be extended to incorporate the pelvis, sparing previously unirradiated areas, though full-dose salvage across large volumes may be challenging [24].

## Discussion

### Patient selection and prescription dose proposal

Candidates for salvage reirradiation must have a good performance status (ECOG 0–2) and no evidence of extensive metastatic disease. They should ideally have a tumour size amenable to BT coverage – often up to ~4–5 cm in diameter – though some larger lesions have been tackled with combined interstitial and intracavitary techniques. Adequate healing capacity and acceptance of possible complications are also critical.

All patients should be evaluated in a multidisciplinary tumour board to ensure optimal treatment selection. BT reirradiation should be considered in patients for whom surgery is contraindicated due to significant comorbidities or in those who explicitly decline surgical management. It is particularly valuable in clinical scenarios where pelvic exenteration is deemed excessively morbid, allowing for a potentially equivalent rate of local control with substantially lower morbidity.

From the perspective of the radiation oncologist, once the above criteria are met, the decision to proceed with reirradiation should follow a detailed pre-planning process. This includes summing the dose contributions from the prior course and estimating the cumulative dose to OARs based on the expected distribution of the new treatment. We recommend considering a prescription dose in the range of 40–47.5 Gy EQD2₁₀ as the minimum necessary to achieve effective tumour control, as supported by the evidence summarised in Table 2.

An interval of at least 12 months between previous irradiation and the new course is often cited as a factor for lower toxicity [25]. Some authors note that patients with intervals under 1 year can still be treated, but with an elevated risk of complications.

### Technique considerations

Modern BT relies heavily on imaging guidance (CT, MRI) to place catheters or needles precisely. Interstitial HDR BT is particularly beneficial for irregular, bulky or lateral recurrences, as the catheters can be placed directly in or around the tumour. Hybrid techniques combine intracavitary (e.g., tandem, vaginal cylinders) with needle implants for better coverage.

Dosimetry commonly focuses on ensuring the prescription dose (e.g., 90% isodose coverage of the CTV) while keeping OAR volumes below defined thresholds. The dose-limiting structures often include the urethra (for vulvar or distal vaginal recurrences), bladder and rectum.

### Outcomes and prognostic factors

In broad retrospective data, salvage BT reirradiation can yield local control rates from about 40% to over 80%, depending on tumour size, total dose and the interval since previous radiation [7–10]. Smaller tumours, higher prescription doses and longer radiation-free intervals tend to correlate with improved outcomes.

Severe (grade 3–4) late toxicities, such as fistulas (rectovaginal, vesicovaginal), necrosis or severe bleeding, remain a risk but can be mitigated by meticulous planning and careful fractionation. Some reports suggest higher toxicities when total doses exceed 40–50 Gy EQD2_10, particularly in previously irradiated patients [7, 15]. However, it is a balance: too low a dose may fail to control the recurrence.

### Comparison with surgery

For central pelvic recurrences, exenteration can be curative. However, exenteration entails extensive morbidity, reduced quality of life and a prolonged recovery. The 5-year OS can range from 10% to 56%, with perioperative morbidity rates of 22%–66% [10, 23]. By contrast, salvage BT can preserve organ function and potentially avoid major surgery, though it must be carefully weighed against the risk of complications in tissues already near tolerance.

### Role of systemic therapy

Concurrent chemotherapy is sometimes added to reirradiation protocols, though there is no universal consensus. Some authors prefer sequential or neoadjuvant chemotherapy [8, 22], aiming to shrink the tumour before BT. Others worry about increased risks of fistula formation, especially when combining radiosensitising chemotherapy with a high-dose local approach. Decisions tend to be individualised.

### Proposed OAR constraints for pelvic reirradiation

In reirradiation scenarios, tolerance of OAR is the primary dose-limiting factor. Because the pelvis has often received substantial prior exposure, cumulative doses can rapidly approach or exceed tissue tolerance. Several authors have proposed guidelines for rectum, bladder, small bowel and urethra based on retrospective analyses of reirradiation in various settings (e.g., BT, SBRT). While there is no universal consensus, the values presented below in Table 1 serve as a useful reference. In practice, clinicians should adapt these constraints to each patient’s prior dose distribution, the time elapsed since previous irradiation and specific technique (e.g., HDR BT versus external beam). In some cases, guidelines originally developed for prostate BT (e.g., GEC-ESTRO ACROP) are adapted cautiously for gynaecological reirradiation, with the understanding that the clinical context differs.

### Practical considerations and future directions

In many institutions, the number of annual reirradiation cases is relatively small [10]. This underscores the importance of centralised care or referral to centers with significant expertise in BT for recurrent disease. Standardising OAR constraints, prescribing guidelines and best practices for needle implant techniques could improve outcomes further.

Ongoing developments in 3D printing have fostered patient-specific applicators, as illustrated in Case 2. Such approaches permit more personalised implantation and coverage of anatomically challenging regions. The routine use of MRI-based planning also refines delineation of the target and healthy structures, potentially reducing margins while ensuring robust coverage.

Prospective trials in this area remain difficult, given the heterogeneity of recurrent disease, prior therapy and patient comorbidities. Nonetheless, collaborative research and registries can help refine dose constraints, define fractionation schedules and identify prognostic factors that predict for cure versus high toxicity.

## Conclusion

Reirradiation with BT is an important salvage approach for local recurrences of gynaecological malignancies in patients who cannot undergo or decline radical surgery. Modern imaging and implant techniques have helped reduce toxicity while improving local control rates, which, in well-selected patients, can be comparable to or better than surgical outcomes.

The two clinical cases presented illustrate the process of careful patient selection, detailed pre-planning to ensure feasibility, implant procedures tailored to each anatomical site and attention to cumulative OAR doses. Despite differences in disease site (vulva versus vaginal vault), both patients tolerated the salvage procedure without severe toxicity. One achieved a complete response at 6 months, while the other had an initial partial response, later requiring additional systemic therapy for progressive disease.

A robust body of retrospective evidence supports the potential curative role of BT-based reirradiation, especially for relatively small, central or paracentral recurrences. Adequate performance status, acceptable prior dose levels and at least 1 year since previous radiation are important selection factors. Adherence to cumulative dose constraints – particularly to the rectum, bladder and urethra – can reduce the risk of life-altering complications like fistulas.

While many questions remain regarding optimal fractionation, total dose and the role of concurrent chemotherapy, BT stands as a vital tool in the armamentarium against recurrent gynaecological cancers. As imaging and treatment planning technology continue to evolve, it is likely that reirradiation with BT will become increasingly safe, precise and effective for the challenging scenario of local relapse in already-irradiated tissues.

## Conflicts of interest

There are no conflicts of interest for any of the authors.

## Funding

No funding was provided for this manuscript.

## Figures and Tables

**Table 1.** Proposed OAR dose constraints in pelvic reirradiation. Dosimetric data from both clinical cases are shown. All doses are shown in EQD2, α/β = 3.

Structure	Suggested constraint	Source/Reference	Case 1		Case 2	
			Total doses for BT	Total doses for EBRT + BT	Total doses for BT	Total doses for EBRT + BT
Rectum	D2cc ≤ 100 Gy	Zolciak-Siwinska *et al* [26]	2.1 Gy	46 Gy	27.3 Gy	63.3 Gy
	D10cc ≤ 110 Gy	Abusaris *et al* [27]	1.5 Gy	45.4 Gy	16.4 Gy	52.4 Gy
	Dmax ≤ 70–110 Gy	Das *et al* [28]	2.6Gy	46.6 Gy	44 Gy	80 Gy
	D2cc ≤ 75 Gy	GEC-ESTRO ACROP [5] (Prostate BT, not reRT-specific)	2.1 Gy	46 Gy	27.3 Gy	63.3 Gy
Bladder	D2cc ≤ 100 Gy	Zolciak-Siwinska *et al* [26]	2.7 Gy	46.7 Gy	30.3 Gy	66.3 Gy
	D10cc ≤ 120 Gy	Abusaris *et al* [27]	2.1 Gy	46 Gy	19.7 Gy	55.7 Gy
	Dmax ≤ 120 Gy	Das *et al* [28]	3.9 Gy	47.9 Gy	62.5 Gy	98.5 Gy
	D0.5cc ≤ 110 Gy	Slevin *et al* [29]	3.2 Gy	47.2 Gy	36.7 Gy	72.7 Gy
	D10% ≤ 120 Gy	GEC-ESTRO ACROP [5] (Prostate BT, not reRT-specific)	2.6 Gy	46.6 Gy	24.7 Gy	60.7 Gy
Urethra	D30% ≤ 105 Gy	GEC-ESTRO ACROP [5] (Prostate BT, not reRT-specific)	12.4 Gy	56.4 Gy	32.6 Gy	68.6 Gy
Small Bowel	D10cc ≤ 110 Gy	Abusaris *et al* [27]	0.5 Gy	44.4 Gy	9.7 Gy	45.7 Gy

BT: brachytherapy. EBRT: external beam radiotherapy. n/e: not evaluated

**Table 2.** Summary of studies on BT-based reirradiation in gynaecologic cancer.

Study (Year)	Type of study	Cancer Type / recurrence	N° of Patients	Prior treatment	Salvage treatment	Oncologic outcomes	Toxicities* (acute/late)	Observations
Randall *et al* [16]	Retrospective single institution study.	Recurrent or second primary gynaecologic malignancies (six recurrent endometrial carcinoma, four recurrent cervical carcinoma, three new primary vaginal carcinomas).	13	HT + EBRT. Previous cumulative dose: 46–80 Gy (endometrial). 50–95 Gy (cervical).	**LDR permanent or temporary ISBT.** **Isotope used:** ^192^Ir (temporary, *n* = 6), ^198^Au (permanent, *n* = 5), ^103^Pd (permanent, *n* = 2). **Endometrial carcinoma**: 40 Gy with ^192^Ir, 30 Gy with ^198^Au. 70–90 Gy with ^103^Pd. **Cervical carcinoma**: 45 Gy with ^192^Ir and 55 Gy with ^198^Au. **Vaginal carcinoma**: 30–35 Gy with ^198^Au and 55 Gy with ^192^Ir.	62% alive after a mFU of 53 months. **LC**: 16% (endometrial), 50% (cervical) and 100% (primary vaginal).	**Acute**: Not specified. **Late**: Rectovaginal fistula 22 months after BT (1 patient).	
Brabham *et al* [17]	Retrospective single institution study (Indiana University).	Recurrent gynaecologic malignancies (15 vagina, 3 cervical, 1 vulva).	19	**Surgery** (*n* = 13). **EBRT** (*n* = 16): Median dose: 47.3 Gy. **LDR BT** (*n* = 14): Median dose: 27.8 Gy. **HDR BT** (*n* = 2): Median dose: 17.8 Gy. **Median previous dose from all radiation modalities**: 67 Gy.	**^198^Au permanent ISBT.** Median prescribed dose: 50 Gy (range, 25–55).	**Complete response:** 94.7%. mFU: 21 months. **LC**: 63.1%. **OS**: 52.6%.	**Acute**: grade three vaginal mucositis at seed site (*n* = 1). **Late**: mild vaginal fibrosis and mucosal thinning (implant site, asymptomatic).	
Mahantshetty et al [13]	Retrospective single institution study (Tata Memorial centre, Mumbai, India).	Recurrent cervical cancer	30	EBRT alone (n = 5). EBRT and BT (n = 25).	HDR ISBT with ^192^Ir. Median delivered dose: 42 Gy. Applicator: Martinez Universal Perineal Implant Template (MUPIT) (n = 24) and Vienna applicator (n = 6).	Complete response: 76.7%. 2-year results: LC: 44%. DFS: 42%. OS: 52%.	Acute: not significant. Late: Grade 3 proctitis and cystitis (n = 3). Grade 3 vaginal fibrosis and stenosis (n = 3). Vesicovaginal fistula (n = 1). Vaginal ulcer (n = 1).	LC higher with doses >40 Gy EQD2 (52% versus 34%; p = 0.05).
Mabuchi *et al* [15]	Retrospective single institution study (Osaka University Hospital, Japan).	Central recurrence of cervical cancer	52	**Post operative EBRT** (*n* = 35). **Definitive EBRT** (*n* = 17).	**HDR ISBT**: 6 Gy × 7 fx (total dose: 42 Gy). **Applicator**: MUPIT.	**Complete response**: 59.6%. **Estimated 5-year** **OS**: 52.6%.	**Grade 3 or 4 toxicity (25%):** 6 rectovaginal and 7 vesicovaginal fistula.	**Tumour size and treatment-free interval:** Poor prognostic factors of postrecurrence survival.
Amsbaugh *et al* [8]	Retrospective single institution study (University of Louisville).	Recurrent gynaecologic cancers (endometrial: 11, cervical: 7, vulvar: 3)	21	**Pelvic EBRT** (*n* = 18): Median cumulative dose: 75–85 Gy (EQD2) **No previous RT** (*n* = 3).	**HDR ISBT alone** (*n* = 11). **HDR ISBT and EBRT** (*n* = 10). Median dose: **HDR** (^192^Ir) 22.5 Gy (3–5 fx BID). **LDR** (^192^Ir): 41.5 Gy. **Applicator**: Syed-Neblett GYN III template.	**1-year results:** **Freedom from LR failure:** 71.5%. **PFS**: 66%. **1-year and 2-year actuarial OS**: 82.2% and 52.5%,	**Grade ≥3 vaginal, urinary and rectal toxicity**: 28.5%, 9.5% and 19%, respectively.	**Tumour size:** Significant predictor of worse PFS and OS.
Kellas-Ślęczka *et al* [9]	Retrospective single institution study (Maria Skłodowska-Curie Memorial Cancer Centre, Poland).	Locally advanced or recurrent vulvar cancer (Group1: 6 locally advanced tumours, stages III-IVA, FIGO 1994 and Group 2: 8 recurrent vulvar cancer).	14	**Group 1**: 5/6 underwent palliative EBRT 20 Gy in 5 fx. 1/6 incomplete radical CRT. **Group 2:** Radical surgery.	**HDR (^192^Ir) ISBT alone or in combination with EBRT**. ISBT dose and fx: e.g. 31.5 Gy in 9 fx (*n* = 2), 32 Gy in 10 fx (*n* = 2), 35 Gy in 10 fx (*n* = 2).	**Group 1:** **1-year** **OS:** 83%. **1-year PFS:** 33%. **Group 2:** **1- and 3-year OS:** 100% and 80%. **1- and 3-year PFS**: 100% and 62.5%.	**Acute**: Vulvar edema and mucositis (percentage and severity not reported). **Late**: Soft tissue necrosis (*n* = 2, group 2).	
Huang *et al* [12]	Retrospective single institution study (London Health Sciences Centre, Western University, Canada).	Recurrent endometrial carcinoma	40	**Prior RT** (*n* = 16): **EBRT alone** (*n* = 5). **ICBT alone** (*n* = 3). **EBRT + ICBT** (*n* = 8). Dose and fractionation: **EBRT**: 45 Gy in 25 fx. **ICBT**: 21 Gy in 3 fx (BT alone) or 15 Gy in 3 fx (EBRT + BT).	**HDR ISBT median dose**: 7 Gy × 3 fx (ranged from 5 to 7.5 Gy × 3 fx).	**2-year results:** **LC**: 60%. **PFS**: 51%. **OS**: 72%.	**Acute**: Grade 1–2 pain (5%), GU (7%), GI (12%), soft tissue (5%) and dermatologic (12%). **Late**: Grade 3–4 in 4 pts (rectal bleeding, fistula and soft tissue necrosis).	Outcomes are similar between previously irradiated and nonirradiated patients.
Feddock *et al* [19]	Retrospective single institution study (Markey Cancer Center, University of Kentucky).	Recurrent pelvic malignancies (endometrial = 12, cervical = 11, vaginal = 9, vulva = 5, fallopian tube = 3, rectal = 1, anal = 1).	42	**Surgery **(*n *= 35): HT= 24, radical vulvectomy= 9, APR = 2. **Pelvic RT alone** (*n *= 11): Median dose: 58.4 Gy EQD2. **Pelvic RT + BT** (*n *= 30): Median dose: 88.8 Gy EQD2. **Vaginal BT alone** (*n *= 1): Dose: 35.7 Gy EQD2.	**Permanent ISBT. Isotope**: ^131^Cs = 46, ^198^Au = 6. **Type of salvage**: **1st salvage: **45 pts with curative intent, 7 with palliative intent. **2nd salvage**: All with curative intent (*n *= 9). **Median doses:** **First salvage**: 45 Gy. **Second salvage:** 40 Gy. **Most common site:** Vaginal cuff.	**LC:** 73% in first salvage and 33% in second salvage. **4-year LC:** 67.4%. **OS:** 52.4% alive at a median follow-up of 16.3 months.	**Acute:** Grade 1–2 in all pts (local postimplant reactions). **Late:** Grade 3–4 (*n* = 7) in the 1st salvage (mucosal/skin necrosis, 2 rectovaginal and 1 enterovaginal fistula). 100% (*n* = 9) with soft tissue necrosis in the second salvage.	
Umezawa *et al* 14	Retrospective single institution study (National Cancer Center, Japan).	Recurrent cervical cancer	18	**Definitive EBRT** (*n* = 4). **Postoperative EBRT** (*n* = 14).	**HDR (^192^Ir) ISBT.** Dose and fractionation: 48 Gy in 8 fx (*n* = 6), 50 Gy in 20 fx (*n* = 3), 24 Gy in 4 fx (*n* = 3), 47.5 Gy in 19 fx in (*n* = 2), 30 Gy in 5 fx (*n* = 1), 36 Gy in 9 fx (*n* = 1), 42 Gy in 7 fx in (*n* = 1), 45 Gy in 9 fx (*n* = 1). **BT BID with a 6-hour interfractional interval.** **Applicator:** Syed-Neblett perineal template.	**Complete response:** 66.7%. **2-year results:** **Local control:** 51.3%. **PFS**: 20%. **OS**: 60.8%.	**Late**: ≥ Grade 2 adverse events were observed in five patients (27.8%). Four vesicovaginal and rectovaginal fistula, one urethra stricture, one radiation proctitis.	Dose/fractionation regimens heterogeneous: Individualised treatment approach.
Martínez-Monge *et al* 7	Prospective Phase II trial (University of Navarra, Pamplona, Spain).	Locally advanced (*n* = 7) and recurrent gynaecologic cancers (43). 25 previously irradiated: 15 cervical, 3 endometrial, 7 vulvovaginal)	50	**EBRT** (*n* = 25): Median dose of 46 Gy (range 45–70 Gy). **BT** (*n* = 9): Median dose of 10 Gy in 2 fx. **Surgery** (*n* = 16). **Chemotherapy** (*n* = 11).	**Previously irradiated:** Surgery and perioperative HDR ISBT (PHDRB). Dose and fractionation: 32 Gy in 8 BID fx for R0 and 40 Gy in 10 BID fx for close/positive margins. CTV determined intraoperatively under direct vision during open laparotomy. **Unirradiated**: Preoperative CRT followed by salvage surgery and PHDRB (R0 and R1 receiving 16 and 24 Gy, respectively).	**14-year results in reirradiated group:** **LC**: 59.6%. **LRC**: 42.6%. **DFS**: 16%. **OS**: 19.2%.	**Reirradiated group:** Grade ≥3 adverse events in 40% (*n* = 10), including 4 fatal events (pelvic bleeding).	**Prospective Phase II trial, median follow-up 11.5 years** After fatal cases, dose was changed to 24 Gy in 6 fx and no Grade ≥3 AE have been observed since then.
Ling *et al *11	Retrospective single institution study (UPMC Hillman Cancer Center).	Recurrent endometrial cancer (vaginal vault recurrence)	22	**Vaginal BT alone** (*n* = 12). **Pelvic EBRT alone** (*n* = 5). **Pelvic EBRT + vaginal BT** (*n* = 5).	**HDR (^192^Ir) BT alone** (*n* = 11). **EBRT** (Median dose: 45 Gy in 25 fx) + **BT** (*n* = 11). **Salvage surgery** (*n* = 5). **Salvage CHT** (Sequential = 5, Concurrent = 3). **BT regimens**: **ICBT**: 30 Gy in 6 fx (*n* = 6). **ISBT**: 25 Gy in 5 fx (*n* = 4), 22.5 Gy in 5 fx (*n* = 2), 27.5 Gy in 5 fx (*n* = 1), 31.7 in 7 fx (*n* = 1).	**3-year results:** **LC**: 66%. **RC**: 76.6%. **DFS**: 40.8%. **OS**: 68.1%. **Median cumulative D2cc (EQD2**): **Bladder**: 72.1 Gy. **Rectum**: 70.6 Gy. **Sigmoid**: 52.7 Gy.	No grade ≥3 acute or late rectosigmoid or bladder toxicities. One patient developed a late grade 3 left ureteral stricture requiring chronic stent placement.	One of the few studies which reports cumulative dose received by OARs.
Isohashi *et al* 31	Retrospective multicenter review (9 facilities in Japan).	Recurrent gynaecologic cancers (cervical: 140, endometrial: 16, vagina: 7, vulva: 2).	165	**EBRT alone** (*n* = 74). **BT alone** (*n* = 15). **EBRT + BT** (*n* = 76).	**ISBT alone** (*n* = 153), **EBRT + ISBT** (*n* = 3), **ICBT alone** (*n *= 7), **ICBT + needles** (*n* = 2). **BT regimens**: 48 Gy in 8 fx (*n* = 73). 42 Gy in 7 fx (*n* = 46). Mean EQD2 at reirradiation: 62.5 Gy (α/β = 10). Mean cumulative EQD2 for overall RT: 146.4 Gy (α/β = 3).	**3-year results:** **Cervical cancer:** **LC**: 61%. **PFS**: 44%. **OS**: 53%. **Endometrial cancer:** **LC**: 70%. **PFS**: 64%. **OS**: 100% (NA). **Vulvovaginal cancer**: **LC**: 43%. **PFS**: 38%. **OS**: 54%.	**Acute**: not specified. **Late**: Grade ≥ 3 in 49 patients (30 %): Ulceration or necrosis of the vaginal wall (*n* = 34) GI toxicity (*n* = 3). Infections (*n* = 3) Vesicovaginal and rectovaginal fistula (*n* = 10) EQD2 (α/β = 3) of 100 and 150 Gy resulted in 18 % and 14 % probabilities of grade 3 toxicities or higher, respectively.	MVA: Interval to reirradiation <1 year: Significant risk factor for: OS, PFS and LC. GTV ≥25 cm^3^: significant risk factor for OS.

Abbreviations: HT, Hysterectomy; EBRT, External beam radiotherapy; RT, Radiation therapy; HDR, High dose rate; LDR, Low dose rate; ISBT, Interstitial brachytherapy; ICBT, Intracavitary brachytherapy; MFU, Median follow up; LC, Local control; PFS, Progression free survival; DFS, Disease free survival; OS, Overall survival; Pts, Patients; Fx, fractions

*All toxicities are reported according to the NCI Common Terminology Criteria for Adverse Events (CTCAE), version 6.0 (2025)
